# Effects of soil chemistry on tropical forest biomass and productivity at different elevations in the equatorial Andes

**DOI:** 10.1007/s00442-012-2295-y

**Published:** 2012-03-14

**Authors:** Malte Unger, Jürgen Homeier, Christoph Leuschner

**Affiliations:** Plant Ecology, University of Göttingen, Untere Karspüle 2, 37073 Göttingen, Germany

**Keywords:** Aboveground biomass, Ecuador, Soil nutrients, Tree growth, Wood production

## Abstract

The dependence of aboveground biomass and productivity of tropical forests on soil fertility is not fully understood, since previous studies yielded contrasting results. Here, we quantify aboveground biomass (AGB) and stem wood production, and examine the impact of soil chemistry on these parameters in mature tropical forest stands of the equatorial Andes in Ecuador. In 80 plots of 0.04 ha at four elevation levels (500, 1,000, 1,500 and 2,000 m a.s.l., total sample area = 3.2 ha), we measured ten important soil chemical parameters, inventoried all trees ≥10 cm dbh and monitored stem diameter growth with dendrometer tapes in 32 plots. Top canopy height and stem density significantly decreased from 500 to 2,000 m, while tree basal area increased and AGB remained invariant (344 ± 17 Mg DM ha^−1^, mean ± SE) with elevation. Wood specific gravity (WSG) showed a significant, but small, decrease. Stem wood production decreased from 4.5 to 3.2 Mg DM ha^−1^ year^−1^ along the transect, indicating a higher biomass turnover at lower elevations. The only soil variable that covaried with AGB was exchangeable K in the topsoil. WSG increased with decreases in N mineralisation rate, soil pH and extractable Ca and P concentrations. Structural equation modelling (SEM) revealed that nitrogen availability acts on stem wood production only indirectly through a negative relation between N mineralisation rate and WSG, and a positive effect of a lowered WSG on stem growth. The SEM analysis showed neither direct nor indirect effects of resin-extractable P on wood production, but a negative P influence on AGB. We conclude that nitrogen availability significantly influences productivity in these Andean forests, but both N and P are affecting wood production mainly indirectly through alterations in WSG and stem density; the growth-promoting effect of N is apparently larger than that of P.

**Electronic supplementary material:**

The online version of this article (doi:10.1007/s00442-012-2295-y) contains supplementary material, which is available to authorized users.

## Introduction

In tropical mountains, tree height and tree growth rates typically decrease with increasing altitude (e.g. Leigh 1975; Bruijnzeel and Veneklaas 1998; Moser et al. 2011), but the underlying causes have not yet been fully understood. A multitude of possible explanations have been proposed focusing either on climatic factors (temperature, drought periods, reduced radiation, persistent leaf wetness, high wind speeds, and others) or on edaphic properties of high-elevation sites (soil water logging, elevated soil acidity in combination with putative Al toxicity, shortage of N or other nutrients, recalcitrant litter with slow decomposition, and others) (see Grubb 1977; Bruijnzeel and Veneklaas 1998; Cavelier 1996; Hafkenscheid 2000; Wilcke et al. 2008; Moser et al. 2011). The role of soil chemistry for the structure and growth of tropical forests at variable elevation was most often investigated from a nutrient limitation perspective testing the hypothesis that tree growth in lowland forests is primarily limited by phosphorus, while nitrogen limitation is thought to be characteristic for tropical montane forests (e.g. Vitousek 1984; Tanner et al. 1998; Hedin et al. 2009). However, other soil properties such as soil organic matter content and decomposition rate have also been found to change with elevation (Cavelier 1996; Marss et al. 1988; Schrumpf et al. 2001), which may also possibly affect tree stature and growth along mountain slopes. Yet, it has been found difficult to disentangle climatic and edaphic effects on the response of tree stature and growth at different elevations because soil chemical properties themselves are largely influenced by climate, namely temperature and rainfall.

The relationship between soil chemical properties (or soil fertility) and forest biomass has been investigated in more detail in several recent studies in tropical lowland forests, which, however, have produced conflicting results. Laurance et al. (1999) and DeWalt and Chave (2004) found a higher aboveground biomass in forests on nutrient-rich Entisols than in forests on nutrient-poor Oxisols, probably reflecting reduced nutrient limitation of tree growth on the richer soils. In contrast, van Schaik and Mirmanto (1985) reported lower biomass in forests on P-rich lowland soils because tree individuals turned over faster in the stand. Other authors found no relationship between soil nutrients and aboveground biomass in tropical forests (e.g. Ashton and Hall 1992), which may indicate that soil chemistry plays only a secondary role in the structure of tropical forests while climatic influences dominate. A better understanding of how forest biomass and tree growth are co-varying with soil nutrient availability is needed to reach more adequate estimates of C stocks in, and C sequestration by, tropical forests than are available today (Paoli et al. 2008).

This study investigates the variability of soil chemical properties, aboveground forest structure and tree growth in a matrix of 80 forest plots on the eastern slopes of the Ecuadorian Andes with the aims (1) to quantify effects of soil variation on stand structure, aboveground live biomass (AGB) and wood production, and (2) to disentangle the interacting effects of forest structure and soil chemical properties on wood production using structural equation modelling (SEM). From the current knowledge on elevational change in tropical forest stature and growth, we predicted that (1) the turnover rate of aboveground biomass (biomass/production) declines with elevation due to a more rapid altitudinal decrease in productivity than in standing biomass, and (2) the productivity decrease along the slope is influenced by the supply of N and P in a complex manner, because the availability of N tends to decrease and that of P to increase with elevation (Vitousek 1984; Unger et al. 2010). The extensive plot matrix of the study reflected the small-scale (plot-level and topographic) variation in soil properties and forest vegetation in a characteristic landscape patch of the equatorial Andes (20 plots per elevation) and also covered the altitudinal variation over an altitudinal distance of 1,500 m (four elevation levels, from lowland forest to lower montane forest). We put a special emphasis on measuring the plant-available fractions of five key plant nutrient elements (N, P, Ca, Mg, K); further, we investigated not only aboveground biomass as a static variable but also stem increment as a productivity parameter.

## Materials and methods

### Study area, plot selection and stand structural analyses

Eighty rainforest plots in the province of Napo, NE Ecuador, were selected for the joint study of soil chemistry, forest structure and aboveground productivity. The study region consists of the Sumaco Biosphere Reserve (SBR) and its direct neighbourhood on the eastern slopes of the equatorial Andes stretching from the foothills in the Amazonian lowlands at about 250 m a.s.l. to the páramo at 3,700 m; the study region includes the Sumaco volcano. The area still harbours large areas of intact forest from lowland to upper montane forests with a very species-rich flora (probably more than 6,000 plant species; Neill and Palacios 1997).

The climate in the region is humid to perhumid with mean annual rainfall exceeding 2,500 mm throughout the area; monthly precipitation never usually drops below 100 mm (Neill and Jørgensen 1999). Even higher rainfall (>4,000 mm year^−1^) is assumed to occur on Sumaco volcano and the Cordillera Guacamayos. Our own temperature measurements in the study period indicate that mean annual temperature decreases from approximately 22.9 °C at 415 m (Jatun Sacha) to 14.3 °C at 2,015 m (Sumaco volcano).

The geology and soils show a considerable heterogeneity throughout the study region covering two distinct physiogeographic regions, the Oriente basin in the Amazon lowlands and the Sub-Andean Zone. The former, quaternary clastic sediments include a variety of deposits, from lavas and pyroclastics of various grain sizes to colluvial/alluvial materials (piedmont fans) and alluvial fills. The Sub-Andean zone is topographically more diverse and consists of foothills rising to elevations of up to 2,000 m and deeply dissected east-flowing rivers. The Sub-Andean zone borders the Cordillera Real and is a back-arc fold-thrust belt tectonically associated with the Andes (Baldock 1982). In most of the area, parent rocks of the Cretaceous dominate, mainly limestone. However, basalt is present at Sumaco volcano and granite at Hakuna Matata; slates are found at Cordillera Guacamayos (Sauer 1971). In general, the geology is variable in the study region but not well explored.

Twenty-five soil pits (4–8 per elevation level) were excavated close to the study plots. Most of the profiles at the 500 and 1,000 m elevation level were classified as Geric Ferralsols according to the World Reference Base for Soil Resources (IUSS Working Group 2006), while Cambisols were dominant at elevations of 1,500 and 2,000 m.

Based on the most recent vegetation classification for Ecuador (Palacios et al. 1999), the species-rich forest stands can be classified as evergreen lowland forests at 500 m, as evergreen premontane forests at 1,000 m, and as evergreen lower montane forests at 1,500 and 2,000 m.

At four elevation levels (500, 1,000, 1,500 and 2,000 m), 20 plots, each of 20 m × 20 m size, were selected in order to include the typical spatial variation in soil types and related forest vegetation at a given elevation. Four different elevations were investigated for covering the variation in soils and vegetation caused by altitude. We studied 80 plots in total with a cumulative forest area of 3.2 ha. Eleven sites belonging to nine different localities (see Table 1; Fig. S1 for further information on the study sites) were selected. A first criterion for site selection was the presence of old-growth forests without any sign of human impact. Since no intact forest is left along the roads throughout the study area, this criterion considerably reduced the number of potential research sites. A second criterion was that a study site should be accessible in a half-day trip from the province capital of Tena for logistical reasons (processing and storage of soil samples). Each site with a size of about 5 km^2^ covered a variety of forest stands that were representative for the respective elevation with its variable topography and geology. The 5–12 permanent plots per site (see Table 1) were selected after carefully exploring the variation in forest vegetation at the respective study site. To reduce the variation in forest structure and tree growth rate that is present within the natural mosaic of different aged forest patches in primary tropical forests, we decided to use a stratified sampling design with all plots selected in parts of the forests that were old-growth, belonged to a late-successional stage according to their species composition, and had a closed canopy.

**Table 1:** The 11 study sites at four elevation levels with elevation range covered, number of study plots (plots equipped with dendrometers in *parentheses*), mean number of stems (dbh ≥ 5 cm), stand basal area (dbh ≥ 10 cm) and mean number of tree species (dbh ≥ 5 cm) per plot (means ± 1SE)

Elevation level (m)	Study site	Elevation range (m)	No. of plots	Stems (400 m^−2^)	Stand basal area (m^2^ 400 m^−2^)	Tree species (400 m^−2^)
500	Jatun Sacha (PR)	400–450	12 (4)	55.5 ± 2.4	1.5 ± 0.1	40.5 ± 2.6
	Selva Viva (PR)	445–520	8 (3)	66.4 ± 3.5	1.8 ± 0.2	49.4 ± 3.2
1,000	Hakuna Matata (PR)	960–1,080	6 (2)	72.3 ± 5.0	1.8 ± 0.1	43.3 ± 4.3
	Cordillera Galeras (NP)	1,050–1,130	9 (3)	50.9 ± 2.9	2.0 ± 0.2	35.2 ± 2.9
	Rio Hollín (PR)	1,165–1,200	5 (3)	75.8 ± 4.2	2.0 ± 0.3	28.6 ± 1.8
1,500	Cordillera Galeras (NP)	1,450–1,600	7 (3)	76.4 ± 6.6	1.7 ± 0.1	40.1 ± 2.5
	Cocodrilos (NP)	1,490–1,570	5 (3)	47.6 ± 3.7	2.2 ± 0.1	28.1 ± 1.2
	Sumaco (NP)	1,580–1,630	8 (3)	41.8 ± 2.1	2.2 ± 0.2	26.4 ± 0.9
2,000	Sumaco (NP)	1,920–2,015	7 (3)	38.3 ± 3.7	2.0 ± 0.2	22.3 ± 2.5
	Cord. Guacamayos (ER)	1,940–2,000	8 (3)	57.7 ± 6.2	1.4 ± 0.1	29.4 ± 3.0
	Yanayacu (PR)	2,055–2,085	5 (2)	41.0 ± 3.7	1.9 ± 0.2	19.0 ± 1.0

The conservation status of the study sites is indicated by *PR* private reserve, *NP* national park and *ER* ecological reserve

The mean distance between the plots of a site varied between 100 m and 1.8 km. Thus, all plots represented independent sampling units each containing a random sample of 16–45 stems ≥10 cm of diameter at breast height (dbh). The plot size (400 m^2^) was small enough to keep environmental factors and forest structure sufficiently homogenous within the stand.

In every plot, we recorded all stems of living trees with dbh ≥ 5 cm. The diameter of all trees (including palms) ≥10 cm was measured and the basal area calculated. Stem density was recorded per 400 m^2^ ground area for all stems ≥5 and ≥10 cm dbh. For species determination, we collected voucher specimens of all unknown species; duplicates were deposited in the herbaria QCA, QCNE and GOET.

### Measurement of stem increment and calculation of aboveground biomass

Stem increment measurements were conducted in two to four randomly selected plots per study site (33–60 % of the plots per site) to equally cover the four elevation levels. In 32 of the 80 plots, all stems with dbh ≥10 cm (*n* = 1,016) were equipped with dendrometer tapes (type D1; UMS, Munich, Germany) that were monitored consecutively for stem diameter growth. The tapes were always mounted at 1.3 m height on the stem; on stems with buttresses or irregular bark surfaces, the measuring point was moved upwards to a height where measurement was possible. The tapes were read about 3 months after installation and subsequently at least once in each subsequent 6-month period; thus, for most of the plots, two or three (or even more) half-year readings during the measuring period November 2005–May 2011 exist. Changes in dbh were determined to the nearest 0.1 mm. Annual diameter increment was calculated by relating the diameter difference between the first and last reading to a full year. The annual cumulative basal area increment per plot was obtained by adding the basal area increments of all trees of a plot. Trees that died during the measuring period and trees that reached the diameter threshold of 10 cm dbh during the measurement period were excluded from the calculation (Clark et al. 2001b).

To calculate aboveground live woody biomass (AGB), we applied the allometric equation of Chave et al. (2005) which was derived for tropical wet forests, with stem diameter, wood specific gravity (WSG) and tree height as parameters. We considered WSG because this variable may have a profound influence on the aboveground biomass of tropical forests (Baker et al. 2004). WSG data for the tree species were obtained from Chave et al. (2006) or, in cases of missing information on species, genera or family means of WSG were calculated from the same source and applied to the respective species. For trees that could not be identified or are still in the process of identification (i.e. for 4.9 % of all stems), we used the mean wood density of the respective plot.

Tree height was measured with a Vertex IV height meter and a T3 transponder (Haglöf, Langsele, Sweden) in April/May 2011 during the last diameter census in a sub-sample of 836 trees (in 29 plots) of the 1,016 trees monitored for diameter growth. From the tree height data, we calculated the mean height of the five tallest trees of a plot as an estimate of top canopy height of the stand. We fitted individual log-linear dbh–height curves (*y* = *a* + *b* ln *x*) for 10 of the 11 study sites (Table S1). These equations were used to estimate the height of all those trees that were examined for dbh but not for height. For the Cocodrilos site at 1,500 m, we used a log-linear dbh–height relationship which is based on the pooled measurements from Sumaco and Galeras at 1,500 m.

We used two different approaches to calculate the aboveground biomass and the stem biomass growth rate (in the following, termed AGB increment) at the plot level. The first method ignored the inclination of the plot terrain and calculated with an uncorrected ground area of 400 m^2^ (following de Castilho et al. 2006), while the alternative considered plot inclination and corrected the actual plot area by dividing plot length by cos α with α being the inclination angle. We present only the uncorrected data of the first approach, because the second approach with inclination correction yielded only slightly different correlations and gave the same significant relationships as the first one.

### Soil sampling and chemical analyses

In order to characterize the study plots in terms of their soil chemistry and nutrient availability, we conducted a set of analyses in all 80 plots with a focus on N supply, plant-available P, Ca, Mg and K, and soil acidity. The analytical methods are described in detail in Unger et al. (2010). Briefly, we extracted each four soil samples per plot in the period April to June 2007. The plots were divided into four equally-sized quadrates of 10 m × 10 m size and the samples were taken in the centre of each quadrate using a soil corer of 5 cm diameter and 25 cm length. The soil core was split into two sub-samples (organic layer material and upper 10 cm of mineral soil). The organic layer included the L, F, and H horizons of variable depth; the transition from the organic H horizon to the mineral soil Ah horizon was arbitrarily set at about 30 % organic matter content using morphological criteria of the substratum for estimating organic matter content. The upper mineral soil consisted of A- and B-horizons with much lower organic matter content than the organic layers themselves. In the laboratory at the University of Göttingen, the following parameters were measured: total carbon and nitrogen (gaschromatography), resin-extractable phosphorus (resin-bag method; Dowex 1 × 8–50), salt-exchangeable K, Mg, Ca, Al (NH_4_Cl percolation with subsequent element analysis by atomic absorption spectroscopy), and soil pH (in KCl). N net mineralisation and nitrification rate (buried bag method) were measured in the field through 8 days of in situ incubation of topsoil material in polyethylene bags and colorimetric NH_4_^+^ and NO_3_^−^ determination at the beginning and end of the exposure period by continuous flow analysis (Cenco/Skalra Instruments, Breda, Netherlands) in the laboratory. Before and after incubation, the NH_4_^+^–N and NO_3_^−^–N concentrations were determined by extracting the samples in K_2_SO_4_ solution (chloroform was added for retarding microbial growth). All extracts were frozen directly after extraction and then transferred to Germany by plane. From the 8-day incubation period, we extrapolated to annual rates of net mineralisation and net nitrification.

Since we assumed a strong influence of the vegetation on the chemical composition of the organic soil layers, all subsequent correlation and SEM analyses focussed on the mineral topsoil and its importance for the vegetation.

### Statistical analyses

Linear regression analyses were applied to identify significant relationships between elevation as independent variable and soil parameters and stand structural and productivity variables. All regressions were calculated using Xact software (version 8.0; SciLab, Hamburg, Germany). To test for significant differences in nutrient concentrations among the plots of different elevations, nonparametric analyses of variance (Kruskal–Wallis test), combined subsequently with two-sample tests (Wilcoxon *U* test) were conducted.

To reduce the number of soil variables and to ensure that the subsequent analyses were not affected by the problem of multi-colinearity, we applied a principal components analysis (PCA) to summarise soil data in five independent axes that together explained 83.8 % of the variation in the soil data. These major axes indicated gradients in organic layer depth, mineral soil pH, total N and C/N ratio (axis Soil PC1), exchangeable K, Mg and Ca in the mineral soil (Soil PC2), topsoil N mineralisation and nitrification rate (Soil PC3), exchangeable Al in the mineral soil (Soil PC4), and resin-exchangeable P in the mineral soil (Soil PC5; see Table S2).

**Fig. 1:**
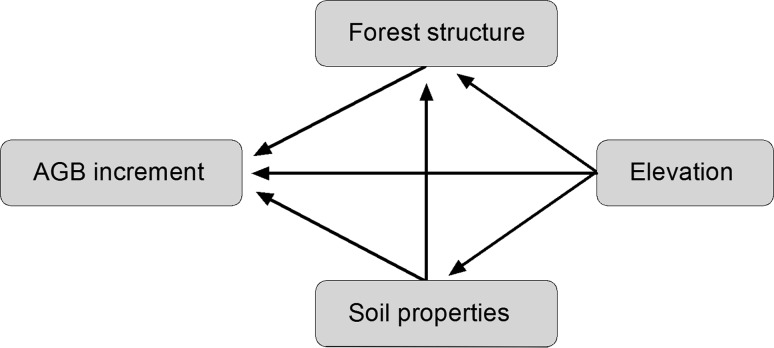
Illustration of plausible interaction pathways in the studied forest ecosystem. Each *arrow* drawn from the two *boxes* *Forest structure* and *Soil properties* indicates that pathways from the respective variables (Forest structure: AGB, stem density, wood specific gravity; Soil properties: PCA axes Soil PC1 to Soil PC5) were included in the initial model

Structural equation modelling was used for identifying the combination of factors that best predicted AGB increment. SEM is an advanced and robust multivariate statistical method designed to provide insight into complex networks of interactions as they are characteristic for the tree growth–environment relationship (Malaeb et al. 2000; Grace 2006). We started with an initial model which contained all plausible interaction paths between the soil chemical, stand structural and productivity-related variables (Fig. 1) based on current knowledge and results of previous studies. AMOS 5.0.1 software (Arbuckle 2003) was used to fit the data to the hypothesized path model and to determine path coefficients using the maximum likelihood method. We assessed the goodness of model fit using the *χ* ^2^ value and the associated *p* value. Since we used SEM in an explorative way, the original model has been subject to modification. We removed insignificant paths one by one to test whether inclusion of those paths in the model significantly increased the *χ* ^2^ value. For all dependent variables, we calculated *R* ^2^ values that indicate the proportion of variance explained by the model.

## Results

### Elevational variation in soil chemical properties

Most of the investigated soil chemical and morphological properties showed a moderate to high variability among the 20 plots per elevation level (Table 2). Significant increases with elevation between 500 and 2,000 m were detected for the thickness of the organic layers (*R* ^2^ = 0.48, *p* < 0.001), the pH_KCl_ value (*R* ^2^ = 0.42, *p* < 0.001), and the resin-extractable phosphorus concentration (*R* ^2^ = 0.20, *p* < 0.001) of the mineral topsoil, while the exchangeable cation concentrations, total N concentration (N_t_) and the C/N ratio of the mineral soil showed no clear elevational trend. Net N mineralisation rate (*R* ^2^ = 0.11, *p* = 0.002) and N nitrification rate (*R* ^2^ = 0.13, *p* < 0.001) both decreased significantly with altitude.

**Table 2:** Means (±1SE) of various soil properties of the organic layers and the mineral soil in each 20 plots at 500, 1,000, 1,500 and 2,000 m elevation in the Sumaco Biosphere Reserve transect

Parameter	Elevation (m)			
	500	1,000	1,500	2,000
Topsoil horizon (organic layers and uppermost mineral soil)				
Depth of org. layers (cm)	1.58 ± 0.21^a^	2.39 ± 0.31^a^	6.34 ± 0.47^b^	5.34 ± 0.32^b^
N net mineralization rate (kg N ha^−1^ year^−1^)	572.61 ± 59.29^a^	310.91 ± 73.08^b^	266.98 ± 53.53^b^	311.02 ± 38.36^b^
N net nitrification rate (kg N ha^−1^ year^−1^)	534.40 ± 52.70^a^	335.85 ± 68.39^ab^	298.99 ± 32.19^b^	280.06 ± 36.40^b^
Mineral soil (0–10 cm)				
pH_(KCl)_	4.08 ± 0.42^a^	4.47 ± 0.54^b^	4.44 ± 0.46^bc^	4.51 ± 0.47^c^
N_t_ (mmol g^−1^)	0.20 ± 0.01^a^	0.51 ± 0.05^b^	0.56 ± 0.03^b^	0.58 ± 0.02^b^
C/N ratio (mol mol^−1^)	10.24 ± 0.19^a^	11.92 ± 0.23^b^	13.11 ± 0.33^c^	11.85 ± 0.19^b^
K_ex_ (μmol g^−1^)	1.72 ± 0.17^a^	2.54 ± 0.16^b^	1.64 ± 0.15^a^	1.86 ± 0.20^a^
Mg_ex_ (μmol g^−1^)	5.70 ± 0.53^ab^	7.56 ± 0.85^a^	4.51 ± 0.56^b^	4.73 ± 0.70^b^
Ca_ex_ (μmol g^−1^)	14.22 ± 2.08^a^	41.28 ± 11.97^a^	21.96 ± 4.10^a^	30.35 ± 7.45^a^
Al_ex_ (μmol g^−1^)	44.85 ± 5.29^a^	57.20 ± 7.76^a^	44.33 ± 3.55^a^	40.48 ± 5.70^a^
P_a_ (μmol g^−1^)	0.10 ± 0.04^a^	0.36 ± 0.08^a^	0.43 ± 0.15^a^	1.20 ± 0.31^b^

The N mineralisation measurements were conducted in 10 cm deep cores of the topsoil horizon consisting of the organic L, F, H layers of variable depth plus the underlying mineral topsoil

* N*_*t*_ total nitrogen;* K*_*ex*_,* Mg*_*ex*_, Ca_*ex*_,* Al*_*ex*_ NH_4_Cl-exchangeable concentrations of* K*,* Mg*,* Ca* and* Al*;* P*_*a*_ extractable P fraction according to the resin-bag method. Different letters indicate significant differences between the elevations. This table was redrawn after Unger et al. (2010)

### Elevational variation in forest structure and aboveground productivity

**Fig. 2:**
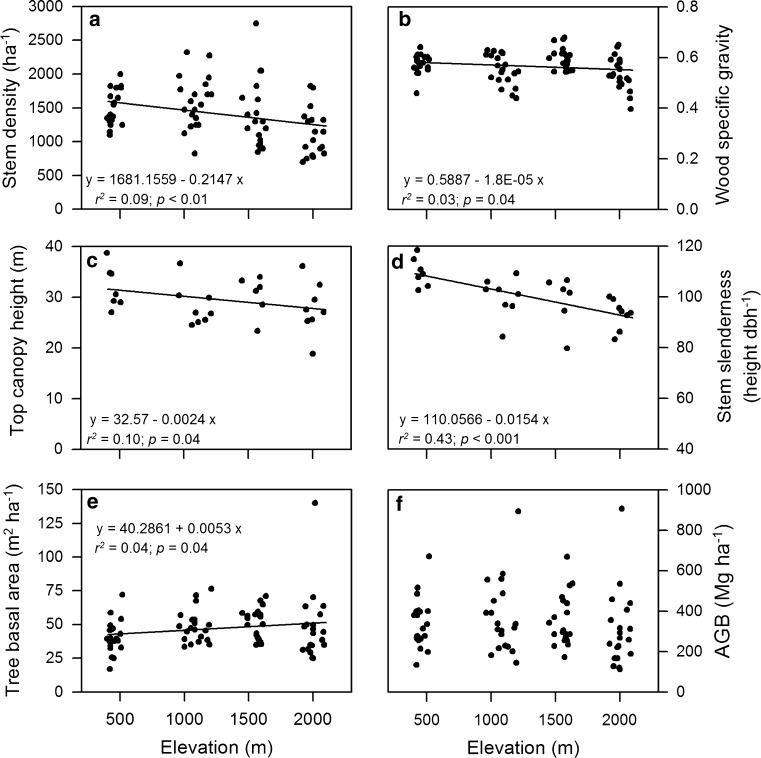
Stem density (**a**), wood specific gravity (**b**), top canopy height (**c**), stem slenderness (**d**), stand basal area (**e**) and above-ground biomass (**f**) as a function of elevation (*n* = 80 study plots, except for top canopy height and stem slenderness with only 29 plots being included). All stems with dbh ≥ 10 cm were considered, except for (**a**) where all stems with dbh ≥ 5 cm were taken into account

The number of tree stems with dbh ≥5 cm varied between 28 and 110 per 400 m^2^ in the 80 studied plots (700–2,750 stems ha^−1^) and seemed to increase slightly from 500 to 1,000 m elevation, but to decrease again higher upslope (Fig. 2a). If only the stems ≥10 cm dbh are considered, the variation among the plots was less pronounced, revealing a maximum at 1,000 m (mean density: 864 stems ha^−1^). The plot means of WSG showed a slight but significant decrease from the 500 m level (0.58 ± 0.01; mean ± 1SE) to the 2,000 m level (0.54 ± 0.01; mean ± 1SE; Fig. 2b). Top canopy height decreased significantly with elevation from 32.0 ± 1.6 m at 500 m to 27.8 ± 1.8 m at 2,000 m (Fig. 2c). The tree height/dbh ratio (stem slenderness) declined significantly (Fig. 2d). Tree basal area (dbh ≥ 10 cm) slightly increased from 500 m (40.8 ± 2.8 m^2^ ha^−1^) to 1,500 m (50.5 ± 2.6 m^2^ ha^−1^) and remained stable higher upslope (Fig. 2e).

Aboveground live tree biomass (dbh ≥ 10 cm) as calculated from dbh, WSG and tree height revealed a considerable scatter among the 20 plots at each of the four elevation levels (100–900 Mg dry mass ha^−1^) with no clear elevational trend visible between 500 and 2,000 m (Fig. 2e). The AGB means of an elevation level ranged between 306 Mg ha^−1^ at 2,000 m and 371 Mg ha^−1^ at 1,000 m (Fig. 2f).

**Fig. 3:**
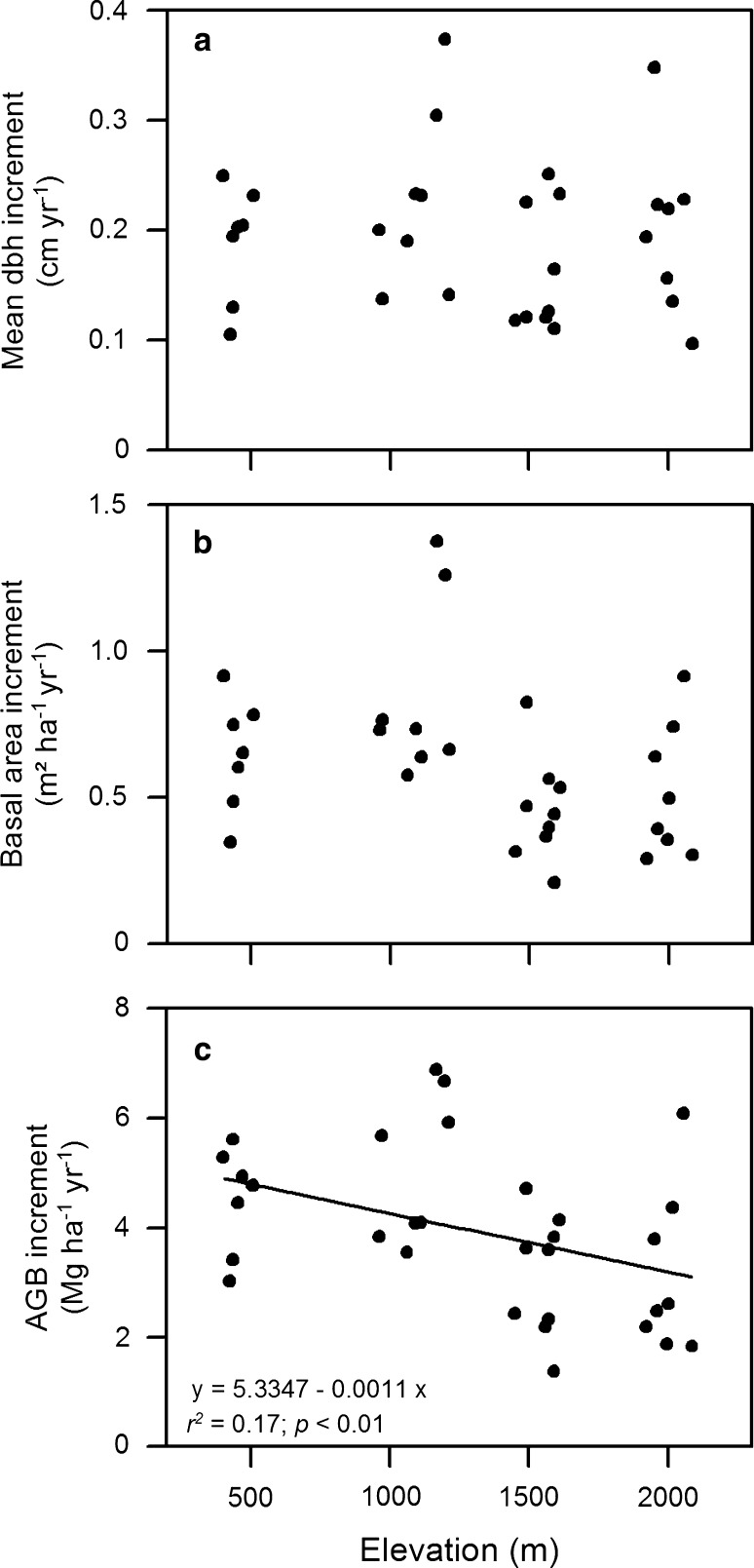
Mean dbh increment (**a**), basal area increment (**b**), and above-ground biomass increment (**c**) of 32 study plots as a function of elevation. All stems with a dbh ≥ 10 cm were considered

Neither the plot-level means of stem diameter increment nor the plot totals of basal area increment showed significant changes with elevation (Fig. 3a, b). However, both growth parameters revealed a large among-plot variation at a given elevation (coefficient of variation for 7–9 plots: 28–44 % for both parameters). The annual increment of stem wood biomass also varied greatly among the stands (1.4–6.9 Mg DM ha^−1^ year^−1^), but showed a significant decrease from the lowland to the lower montane stands (Fig. 3c).

**Fig. 4:**
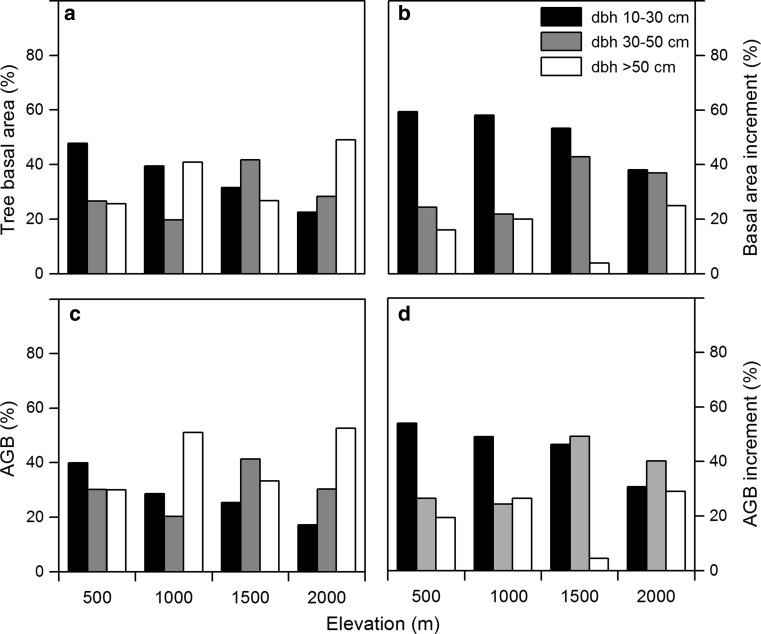
Percental contribution of three different dbh classes of trees (10–30 cm, 30–50 cm, >50 cm) to the stand totals of basal area (**a**), basal area increment (**b**), above-ground biomass (**c**) and AGB increment (**d**) at the four elevation levels (*n* = 80 plots for **a** and **c**; *n* = 32 for **b** and **d**)

Our data revealed no marked elevational trend in the relative contribution of large or small dbh classes to the stand totals of basal area or AGB (Fig. 4a, c). As for the biomass components, we found no elevational change in the relative contribution of small- and large diameter trees to the stand total of stem wood increment (Fig. 4b, d).

### Relationships between soil chemistry and forest structure and productivity

The regression analyses indicated that stem density and WSG both were negatively correlated with pH_KCl_, exchangeable Ca and resin-extractable P (Table 3). In addition, WSG significantly decreased with growing N_t_ concentrations of the mineral soil and increasing nitrification rates in the topsoil. Not only WSG increased with a deterioration of the soil nutrient status (N, P, Ca and pH) but also top canopy height of the stands.

**Table 3:** Results of correlation analyses on the influence of elevation and 11 soil parameters (source) on eight stand structural and productivity variables determined in permanent plots at four elevations levels (see Table 1)

Source	Stem density (ha^−1^)	Top canopy height (m)	Basal area (m^2^ 400 m^−2^)	AGB (Mg ha^−1^)	WSG (g cm^−3^)	DBH increment (cm year^−1^)	BA increment (m^2^ ha^−1^ year^−1^)	AGB increment (Mg ha^−1^ year^−1^)
Elevation	−0.31**	−0.32*	0.19*	**−**0.07	−0.19*	**−**0.03	**−**0.29	−0.42**
Topsoil horizon (organic layers and uppermost mineral soil)								
Depth of org. layers	**−**0.13	**−**0.16	0.05	**−**0.13	0.04	**−**0.03	**−**0.22	−0.34*
N mineralisation rate	**−**0.07	0.12	0.03	0.06	−0.19*	0.13	0.22	0.34*
N nitrification rate	**−**0.12	0.20	0.09	0.06	−0.23*	0.22	0.28	0.40*
Mineral soil (0–10 cm)								
pH_KCl_	−0.43***	**−**0.26	0.11	**−**0.17	−0.28**	0.18	**−**0.11	**−**0.24
N_t_	**−**0.17	−0.31*	0.16	**−**0.03	−0.29**	**−**0.01	**−**0.01	**−**0.10
C/N ratio	0.21*	**−**0.26	0.02	**−**0.04	0.18	**−**0.22	**−**0.12	**−**0.14
K_ex_	0.07	0.06	0.17	0.28**	**−**0.14	−0.34*	**−**0.01	**−**0.02
Mg_ex_	0.01	0.18	0.05	0.16	**−**0.15	**−**0.23	**−**0.01	**−**0.05
Ca_ex_	−0.32**	**−**0.07	0.05	**−**0.06	−0.43***	**−**0.24	**−**0.21	**−**0.27
Al_ex_	0.28**	**−**0.09	0.05	0.08	**−**0.06	**−**0.20	0.51**	0.47**
P_a_	−0.43***	−0.32*	**−**0.02	**−**0.10	−0.46***	**−**0.17	−0.31*	−0.38*

Significant relationships are printed in bold (*n* = 80 for the static data, *n* = 32 for the increment data and *n* = 29 for top canopy height). For units of source variables, see Table 2

Pearson correlation coefficients are shown, plus significance levels (* p ≤ 0.05; ** *p* ≤ 0.01; *** *p* ≤ 0.001)

Aboveground biomass increment increased significantly with the N supply rate (N net mineralisation and nitrification rates) and decreased with organic layer depth. In addition, the regression analysis showed an unexpected positive correlation of exchangeable Al and a negative correlation of resin-extractable P with stem wood production.

**Fig. 5:**
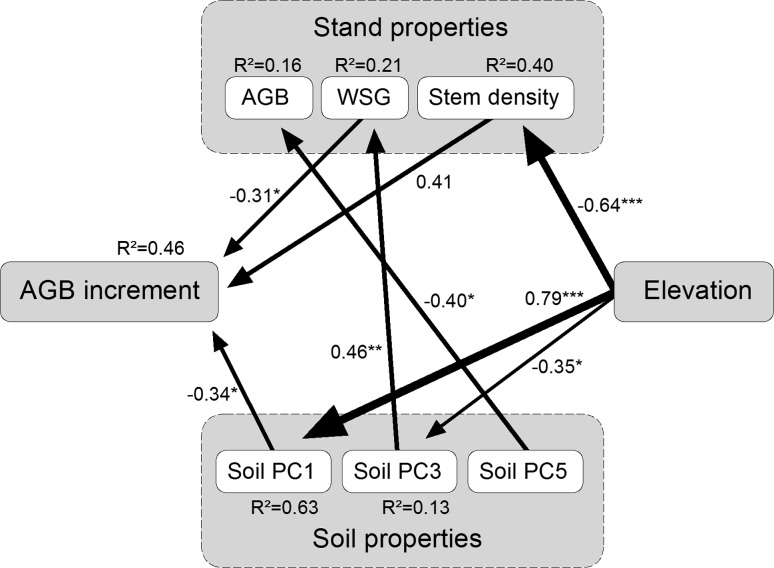
Final structural equation model (*χ* ^2^ = 25.5, 20 *df*, *p* = 0.18) with standardised path coefficients. The size of the *arrows* is proportional to the strength of the path. The significance of the paths is indicated as follows: *p < 0.05, **p < 0.01, ***p < 0.001

The model developed in the SEM analysis explained 46 % of the observed variation in AGB increment (*χ* ^2^ = 25.5, *df* = 20, *p* = 0.18; Fig. 5). Table 4 summarises the total and the direct and indirect effects of various stand structural and edaphic factors on AGB increment as identified by the SEM analysis. In the final model of Fig. 5, all non-significant paths had been eliminated and two soil axes (Soil PC2 and Soil PC4) dropped, which significantly improved the model. Accordingly, only stem density had a positive (total) effect on AGB increment, whereas WSG, the axis Soil PC1 (which includes organic layer depth, pH and C/N ratio) and elevation all had negative total effects. The effect of axis Soil PC3 (which includes N supply) on AGB increment was an indirect one. The strongest effect on AGB increment was exerted by elevation, but this factor acted only indirectly through three different paths. AGB itself had only an insignificant effect on AGB increment (standardised path coefficient = 0.16; path eliminated). Remarkably, the model showed a negative effect of resin-extractable P (included in the Soil PC5 axis) on AGB.

**Table 4:** Standardised direct, indirect and total effects of various stand structural and soil chemical parameters on the AGB increment of 1,016 trees (in 32 plots) according to a SEM analysis

Factors	Direct	Indirect	Total
AGB			
WSG	−0.31		−0.31
Stem density	0.41		0.41
Elevation		−0.48	−0.48
Soil PC 1	−0.34		−0.34
Soil PC 3		−0.14	−0.14
Soil PC 5			

Standardised path coefficient are shown

## Discussion

### Variation in forest aboveground structure

Our investigation in 80 tropical forest plots at lowland and lower montane elevation revealed a much larger scatter of the stem density and AGB data than of the basal area data across the studied edaphic and elevation gradients. Based on the WSG and height data of the trees, we calculated AGB means of 343, 371, 354, and 307 Mg DM ha^−1^ for the 500, 1,000, 1,500, and 2,000 m elevation levels, respectively. Because we avoided early successional stages and canopy gaps during plot selection, our AGB calculation most likely overestimates aboveground biomass on the landscape level. This is indicated when comparing our mean AGB value of the 80 plots (344 Mg DM ha^−1^) to literature data from Central Amazonian lowland forests, for which AGB values in the range of 200–360 Mg ha^−1^ (peak values of 320–360 Mg ha^−1^) have been reported (de Castilho et al. 2006; DeWalt and Chave 2004; Baker et al. 2004; Laurance et al. 1999; Malhi et al. 2006). The influence of the absence of gaps is also evident from the relatively high stand basal area in our sample, when compared to average values between 25 and 35 m^2^ ha^−1^ reported by two large surveys in Amazonian old-growth forests (Baker et al. 2004; Malhi et al. 2006). According to the analysis of Keeling and Phillips (2007), the tropical forests of the world typically do not have AGB values higher than 350 Mg ha^−1^; a notable exception are South-east Asian dipterocarp forests with AGB values of more than 450 Mg ha^−1^ (e.g. Slik et al. 2009, 2010; Borneo).

In contrast to several other transect studies in tropical mountains (e.g. Weaver and Murphy 1990; Raich et al. 1997; Kitayama and Aiba 2002; Leuschner et al. 2007; Girardin et al. 2010; Moser et al. 2011), we did not find a significant altitudinal trend in AGB between 500 and 2,000 m elevation in NE Ecuador. Despite a significant reduction in stem density, stand basal area slightly increased between 500 and 2,000 m along our transect (from about 40 to 50 m^2^ ha^−1^), thereby compensating for the negative effect of lowered stand height on AGB along the slope. A missing elevation effect on AGB was recently also reported by Culmsee et al. (2010) for an altitudinal transect in pre-montane to upper montane forests on Sulawesi, where members of Fagaceae and southern hemisphere conifers with high biomasses play a significant role at higher elevations. A corresponding phylogeographic explanation cannot be given for the NE Ecuadorian transect. Furthermore, the relative abundance of tree individuals in different diameter classes did not vary with elevation which makes altitudinal shifts in demographic patterns unlikely.

### Variation in stem wood production

Comparative productivity measurements in large plot samples have only rarely been conducted in tropical forests because of the considerable labour effort required. We measured stem diameter increment in more than 1,000 trees in 32 plots and calculated basal area increment and wood mass production as indicators of the variation of aboveground productivity in forest stands across a range of edaphic and elevation conditions. Clearly, stem wood increment is only one component of the aboveground biomass production of trees, besides the production of leaf mass, flowers and fruits. The latter components may contribute with more than 50 % to aboveground productivity in tropical forests (Clark et al. 2001a; Girardin et al. 2010), but they could not be measured in the 32 plots of our study. Thus, our coarse wood production data allow the comparison of the carbon sequestration in ‘slow’ C pools such as wood biomass, but they may only give an incomplete picture of the variation in aboveground net primary production across the stands. Due to the small size of our plots (400 m^2^) and the relatively short dendrometer monitoring intervals, we ignored tree mortality in our productivity calculations (following Clark et al. 2001b). Further, we neglected the productivity of the understorey vegetation which typically comprises less than 3 % of the stand’s AGB in tropical moist forests (Brown 1997). With this approach, we found an almost fivefold variation in annual wood biomass production among the 7–9 stands at a given elevation level (1.4–6.9 Mg DM ha^−1^ year^−1^), which may reflect local differences in topography, soil fertility, soil moisture, stand structure and demography, or species composition. Wood increment decreased between 500 and 2,000 m elevation concurrently with the decline in mean air temperature by about 8.6 K from a mean of 4.5 ± 0.4 Mg ha^−1^ year^−1^ at 500 m to a mean of 3.2 ± 0.6 Mg ha^−1^ year^−1^ at 2,000 m. The observed significant leaf area index (LAI) decrease with elevation in our transect (by about 1.1 m^2^ m^−2^ per 1,000 m, i.e. from 6.6 at 500 m to 5.2 at 2,000 m; Unger et al. 2012) is in agreement with the productivity decrease.

A similar range of variation in stem wood production (1.6–5.6 Mg ha^−1^ year^−1^) and a corresponding decrease with elevation has been reported by Girardin et al. (2010) from a forest transect (200–3,000 m) in the Peruvian Andes. Our lowland values are in the lower range of coarse wood productivity values (3.0–11.0 Mg ha^−1^ year^−1^) reported by Malhi et al. (2004) for Neotropical lowland forests. This is not surprising, since we omitted fast-growing early-successional stands in our set of plots. However, the values from our uppermost site are notably higher than the 1.6 Mg ha^−1^ year^−1^ estimated by Girardin et al. (2010) at 2,020 m.

The measured annual wood increment equalled an average relative growth rate of 1.5 ± 0.2 % when scaled to the standing aboveground biomass which agrees well with results reported by Clark et al. (2001a), who assumed that AGB increment should be about 1–2 % of standing biomass in tropical old-growth forests.

### Relationships with soil nutrients

Our study revealed significant relationships between forest structure and productivity, and several parameters characterising soil nutrient availability. Three of the static parameters of forest structure, stem density, top canopy height and WSG, showed significant correlations with those soil chemical conditions that stand for a relatively low fertility and less favourable growth conditions (low pH, low total N and low resin-extractable P), and these three structural parameters were declining with elevation. A negative correlation of WSG with soil fertility has also been documented from Borneo (Slik et al. 2010) and from the Amazon (Baker et al. 2004). In contrast, the other two investigated structural parameters of stand basal area and AGB were only slightly influenced by the nutrient status of the mineral topsoil according to the single-factor correlation analyses (except for a positive effect of exchangeable K on AGB).

With respect to the examined productivity parameters, we obtained evidence that AGB increment is related to the availabilities of N and P in a complex manner as predicted by our second hypothesis. The correlation analysis showed that AGB increment was closely related to N supply (N mineralisation and nitrification rate in the topsoil), while it was negatively associated with organic layer depth. However, the SEM analysis revealed that nitrogen availability apparently acted on stem growth only indirectly through a negative relationship between N supply and WSG and a positive effect of a lowered WSG on AGB increment as indicated by the interaction paths between Soil PC3, WSG and AGB increment in the structural equation model. Soil C/N ratio had a negative effect on AGB increment which is further evidence for a dependence of stem wood production on N availability. Remarkably, the SEM analysis showed neither a direct nor an indirect effect of resin-extractable P (included in Soil PC5 in Fig. 5) on AGB increment and a negative P effect on standing aboveground biomass. The correlation analysis showed a significant negative effect of P on AGB increment, which contrasts with the results found for N. Thus, we conclude that WSG seems to play a central role in the soil fertility–productivity relationship in these forests with N and P both acting mainly indirectly on AGB increment via alterations in WSG and also in stem density. However, the evidence from the SEM analysis suggests that the overall growth-promoting effect of N should be larger than that of P in our transect from upper lowland to lower montane elevation.

Only a few studies have investigated the dependence of the stand structure and productivity of tropical forests on soil nutrient availability. Our results are partly in agreement with the findings of Wilcke et al. (2008) in a south Ecuadorian montane forest where the N_t_ concentration in the organic layers (together with the lignin concentration) was identified as the soil chemical variable with the largest influence on tree diameter growth. In this study, the total P and cation concentrations of the organic layers, and the mineral soil base saturation, had only a small effect on stem growth. However, the study by Wilcke et al. (2008) did not provide data on N mineralisation rate or plant-available P. In a Bornean lowland forest, Paoli et al. (2008) found significant relationships between extractable P and exchangeable K in the topsoil and AGB (in particular for the tallest emergent trees), while the N_t_ influence was not significant. These authors investigated biomass but not growth parameters. While there is no positive influence of resin-extractable P on AGB in our study area, the importance of extractable K for aboveground biomass is in agreement with our results.

Compared to other tropical mountains (e.g. Grubb 1977; Kitayama and Aiba 2002; Moser et al. 2011; Wilcke et al. 2008), our study region in the northeast Ecuadorian Andes seems to be characterised by relatively fertile soils as is indicated by rather small soil C/N ratios, elevated cation and N_t_ concentrations, comparatively thin organic layers and relatively high foliar N and P contents (means of 26 mg N g^−1^ and 1.2 mg P g^−1^ at 2,000 m; unpublished data). Thus, it may well be that the nutrient effect on tree growth is more pronounced in tropical regions with less fertile soils.

For forests at higher elevations, the hypothesis of Vitousek (1984) predicts a shift from P to N limitation of tree growth because soil biological activity and decomposition rate should be lower in the cooler climate of mountain forests, reducing N mineralisation rate. In our dataset, however, a positive effect of resin-extractable P on wood increment was not detected, even though the mean P_a_ concentration in the topsoil increased more than tenfold from 500 to 2,000 m, while the influence of N supply on AGB increment persisted from lowland to montane elevation which seems to contradict this hypothesis.

Even though our data indicate a nutrient (predominantly N) effect on forest structure and productivity, they do not prove it. Other co-varying environmental factors, notably soil moisture, could just as well have an influence (Leuschner et al. 2007; Slik et al. 2010), but were not investigated. In a similar manner, the observation of Paoli et al. (2008) in western Kalimantan that the tallest trees are found on nutrient-rich alluvial soils does not necessarily reflect a nutrient effect, but could perhaps also be the expression of a lower intensity of soil droughts in the alluvial soils, which would support tree height growth.

Fertilisation or moisture manipulation studies, or correlative studies with a more complete set of environmental variables investigated, would be needed to draw more safe conclusions on the role of soil nutrient availability for the structure and functioning of tropical forests. Because studies investigating the co-variation of biomass and productivity with both soil fertility and soil moisture are lacking so far for tropical forests, this question cannot be answered satisfactorily.

We conclude that tropical forest soils on the eastern slope of the equatorial Andes are characterised by a large spatial heterogeneity in the plant availability of the five key nutrients, i.e. resin-extractable P, exchangeable Ca, Mg and K concentrations, and inorganic N supply (N mineralisation and nitrification rate). According to the analysis by Unger et al. (2010), these parameters were more variable across topographic and pedologic gradients at a given elevation than across our 1,500-m elevation transect.

Stem density, top canopy height, WSG and ABG respond significantly, but not congruently, to the soil chemical conditions. AGB, which may be influenced by tree longevity, co-varied with the exchangeable concentration of K in the mineral topsoil. The availability of N (characterised by the in situ N mineralisation and nitrification rates, the soil C/N ratio, and N_t_ concentration) is an important factor with an apparent influence on forest biomass and productivity at both lowland and lower montane elevation; most likely, this relationship is a multi-path interaction. Our results are not fully in agreement with the prediction that N limitation of tree growth should increase with elevation in tropical mountain forests (Vitousek 1984; Hedin et al. 2009).

One of the more remarkable findings is that there is no significant decrease of AGB with elevation, which indicates that old-growth forests can reach similar AGB values from lowland to lower montane elevation despite the fact that stand height and coarse wood productivity are reduced with elevation. A decreasing AGB increment but constant aboveground biomass with elevation indicates a higher biomass turnover at lower compared with higher elevations as was predicted by our first hypothesis.

A decreasing LAI with elevation (as reported by Unger et al. 2012) suggests that litter production decreases in parallel with stem wood production in this transect. Smaller amounts of litter supplied, but a larger organic layer depth, at higher elevations indicate that soil biological activity and thus nutrient supply rates are in general decreasing with elevation as is supported by our N mineralisation data.

Since the studied soil parameters only partly explain the variation of stand structural and productivity parameters, we conclude that other environmental factors such as soil moisture may also exert a major influence on these traits and that manipulation experiments would be necessary to prove causal dependencies along elevational gradients in tropical mountains.

## Electronic supplementary material

Below is the link to the electronic supplementary material.

Supplementary material 1 (DOC 570 kb)

Supplementary material 2 (DOC 54 kb)
