# Supplementary material for: Effects of soil chemistry on tropical forest biomass and productivity at different elevations in the equatorial Andes
Source: Oecologia. 2012 Mar 14;170(1):263–74. doi: 10.1007/s00442-012-2295-y (PMC3422456; doi:10.1007/s00442-012-2295-y)
Supplement: Supplementary file 1 — Supplementary material 1 (DOC 570 kb) [file 442_2012_2295_MOESM1_ESM.doc]

**Effects of soil chemistry on tropical forest biomass and productivity at different elevations in the equatorial Andes**

Malte Unger, Jürgen Homeier & Christoph Leuschner

SUPPLEMENTARY MATERIAL


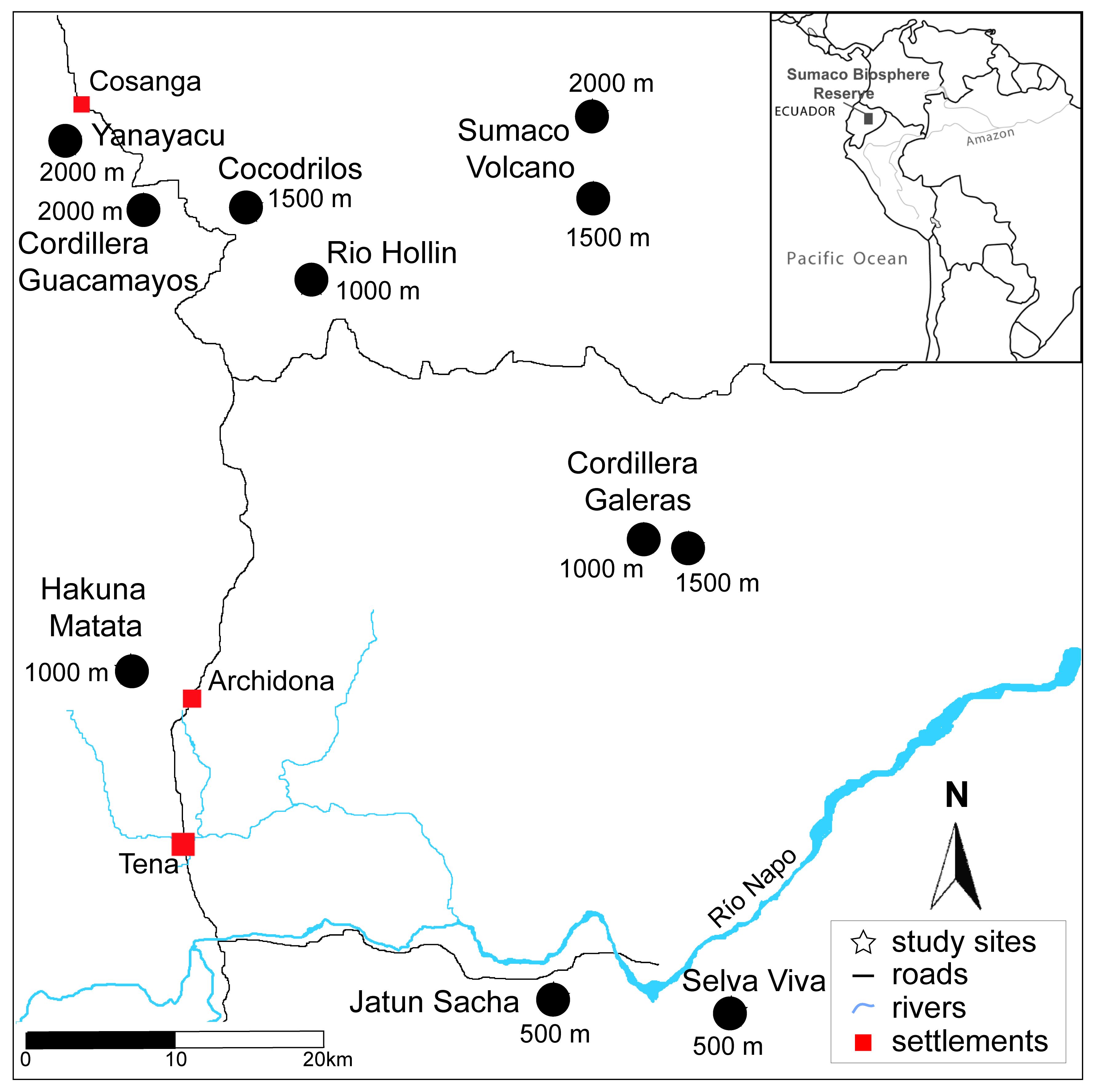


**Fig. S1** Map of the eleven study sites in the area of the Sumaco Biosphere Reserve in NE Ecuador. In total, 80 plots at 11 sites were studied. The number of plots per study site is given in Table 1.
