# Supplementary material for: Effects of soil chemistry on tropical forest biomass and productivity at different elevations in the equatorial Andes
Source: Oecologia. 2012 Mar 14;170(1):263–74. doi: 10.1007/s00442-012-2295-y (PMC3422456; doi:10.1007/s00442-012-2295-y)
Supplement: Supplementary file 2 — Supplementary material 2 (DOC 54 kb) [file 442_2012_2295_MOESM2_ESM.doc]

**Effects of soil chemistry on tropical forest biomass and productivity at different elevations in the equatorial Andes**

Malte Unger, Jürgen Homeier & Christoph Leuschner

SUPPLEMENTARY MATERIAL

| **Table S1** Log-linear dbh-height relationships for ten of the 11 study sites based on the tree height measurement at the respective site. | | | | | | |
| --- | --- | --- | --- | --- | --- | --- |
| **Site** | **Elevation level** | **No. of trees measured** | **dbh range** | **relationship** | **R²** | **p** |
| Jatun Sacha | 500 | 135 | 10.0 - 76.4 | y = -17.4047 + 12.9822 * ln(dbh) | 0.6217 | <0.001 |
| Selva Viva | 500 | 121 | 10.0 - 86.6 | y = -13.5771 + 11.1313 * ln(dbh) | 0.6037 | <0.001 |
| Cord. Galeras | 1000 | 74 | 10.3 – 112.0 | y = -6.5793 + 7.7721 * ln(dbh) | 0.5208 | <0.001 |
| Hakuna Matata | 1000 | 82 | 10.6 – 74.8 | y = -14.1373 + 12.9822 * ln(dbh) | 0.5448 | <0.001 |
| Rio Hollin | 1000 | 125 | 9.8 – 174.1 | y = -14.3645 + 10.9829 * ln(dbh) | 0.6149 | <0.001 |
| Cord. Galeras | 1500 | 93 | 10.0 – 55.0 | y = -9.5812 + 9.4135 * ln(dbh) | 0.501 | <0.001 |
| Sumaco | 1500 | 60 | 10.2 – 156.0 | y = -14.0625 + 10.7945 * ln(dbh) | 0.561 | <0.001 |
| Sumaco | 2000 | 61 | 10.7 – 196.7 | y = -11.1814 + 10.1115 * ln(dbh) | 0.4989 | <0.001 |
| Cord. Guacamayos | 2000 | 63 | 10.7 – 56.4 | y = -17.1346 + 10.7484 * ln(dbh) | 0.557 | <0.001 |
| Yanayacu | 2000 | 49 | 11.6 – 68.8 | y = -17.0356 + 11.5021 * ln(dbh) | 0.7294 | <0.001 |

| **Table S2** Results of a principal components analysis (PCA) on the importance of soil chemical and morphological parameters (see Table 2) conducted for important soil properties of the topsoil and the mineral soil in 80 forest stands of NE Ecuador. Given are the five principal components identified (the amount of explained data variance in brackets) and the related factor loadings. The total explained variance of the five PCs is 83.8%. The most characteristic variables (according to their loading) of each PCA axis are printed in bold. | | | | | |
| --- | --- | --- | --- | --- | --- |
|  | Eigenvectors | | | | |
|  | PC 1  (27.7%) | PC 2  (20.4%) | PC 3  (14.2%) | PC 4  (13.7%) | PC 5  (7.8%) |
| **Topsoil variables** |  |  |  |  |  |
| Depth of organic layers | **0.744** | -0.376 | -0.068 | 0.194 | -0.204 |
| N net mineralization rate | -0.639 | -0.212 | **-0.676** | 0.059 | -0.154 |
| Net nitrification rate | -0.640 | -0.281 | **-0.653** | 0.086 | -0.172 |
| **Mineral soil variables** |  |  |  |  |  |
| pH_KCl_ | **0.706** | 0.004 | -0.262 | 0.453 | -0.139 |
| N_t_ | **0.719** | -0.105 | -0.474 | -0.310 | 0.004 |
| C/N-ratio | **0.660** | -0.345 | -0.030 | -0.443 | -0.305 |
| K_ex_ | 0.012 | **0.639** | -0.088 | -0.590 | -0.082 |
| Mg_ex_ | -0.073 | **0.851** | -0.015 | -0.133 | -0.277 |
| Ca_ex_ | 0.262 | **0.740** | -0.380 | 0.213 | -0.155 |
| Al_ex_ | -0.022 | -0.280 | -0.236 | **-0.717** | 0.256 |
| P_a_ | 0.387 | 0.308 | -0.411 | 0.180 | **0.691** |
|  |  |  |  |  |  |
